# Epidemiology is a science of high importance

**DOI:** 10.1038/s41467-018-04243-3

**Published:** 2018-05-07

## Abstract

Epidemiology dates back to the Age of Pericles in 5th Century B.C., but its standing as a ‘true’ science in 21st century is often questioned. This is unexpected, given that epidemiology directly impacts lives and our reliance on it will only increase in a changing world.

Epidemiology identifies the distribution of diseases, factors underlying their source and cause, and methods for their control; this requires an understanding of how political, social and scientific factors intersect to exacerbate disease risk, which makes epidemiology a unique science. Nevertheless, its definition as a science is debated; among the criticisms of the field are that epidemiology is an inexact science that it is simply a set of tools used by other disciplines, and that its dependence on observational data makes it a form of journalism rather than a science^1,2^. *Nature Communications* editors have visited established epidemiologists and also found, to our surprise, that their impression from the rest of the scientific community is often that epidemiology is not viewed as a ‘true’ science.

Among the many reasons why its scientific significance is sometimes trivialised is its intersection with the so-called ‘soft’ sciences, which have traditionally been thought of as less exact than other disciplines because of their focus on variables that are complex and difficult to quantify, such as human behaviours and interactions. But socioeconomic and lifestyle factors, and features of the built environment, are known to affect health outcomes, including in individuals with cardiovascular^3^ and genetic diseases^4^, and so they cannot be overlooked in studies of human health^5^.

Furthermore, there are tangible results from epidemiological research. It is unquestionable that the discipline has saved millions of lives, from both infectious and non-communicable diseases, through interventions and preventative programs that have been implemented as a result of study findings. In fact, the CDC credits medical epidemiologists with adding 25 years to the average life expectancy of people living in the United States since 1947^6^.

While the exact number of people whose lives have been saved by epidemiological research may not be possible to calculate, its importance in enhancing life quality and longevity cannot be overlooked. Even more significantly, despite the uncertainty, the incompleteness of models and the imperfections of data, epidemiology continues to be at the forefront of saving lives today through forecasting epidemics and pandemics, and identifying diseases likely to cause outbreaks in the future and implementing forward-planning, targeted and collaborative interventions to minimise fatalities^7,8^.

Increasingly, epidemiology is the key to understanding the impact of climate change on disease burden through the effect of temperature, humidity and seasonality on infectious disease dynamics, and the expansion of the ranges of disease vectors. Unlikely to be an isolated case, the State of Texas has reported transmission or outbreaks of Ebola, chikungunya, West Nile, and Zika virus infections within the past 5 years, and this is believed to be attributed to both climate change and rapid population expansion and urbanisation^9^.

Along with increased inequality, and urbanisation, climate change presents new challenges for global health programmes; in light of these, epidemiological research is sure to remain a cornerstone in guiding public health policies in the near future^10^.

So, epidemiology is important but is it a science? Yes, it is. While it may not be helpful to compare it with, say, mathematics, it is a bona fide multidisciplinary approach to the study of human health and disease that follows the scientific method of systematic observation, and the formulation, testing, and modification of hypotheses. If anything, epidemiology is a highly complex science because it needs to consider multiple variables associated with human diseases, such as pathogens, human social or travel dynamics, and the climate. This can mean that the results obtained for a disease and/or outbreak may not always be replicable for the same disease in a different environment.

*Nature Communications* editors appreciate the importance of epidemiology and would like to encourage submissions from the field, especially when applied to tackling issues of public health.
